# Sex-specific associations between self-reported sleep characteristics and 10-year cardiovascular disease risk in men and women of African descent living in a low socioeconomic status environment

**DOI:** 10.1016/j.sleepe.2024.100091

**Published:** 2024-07-08

**Authors:** Philippa E. Forshaw, Arron T.L. Correia, Laura C. Roden, Estelle V. Lambert, Brian T. Layden, Sirimon Reutrakul, Stephanie J. Crowley, Amy Luke, Lara R. Dugas, Dale E. Rae

**Affiliations:** a Health through Physical Activity, Lifestyle and Sport Research Centre and Division of Physiological Sciences, Department of Human Biology, Faculty of Health Sciences, University of Cape Town, Cape Town, South Africa; b Research Centre for Health and Life Sciences, Coventry University, Coventry, UK; c Division of Endocrinology, Diabetes and Metabolism, University of Illinois Chicago, Chicago, IL, USA; d Jesse Brown Veterans Affairs Medical Center, Chicago, IL USA; e Department of Psychiatry & Behavioral Sciences, Biological Rhythms Research Laboratory, Rush University Medical Center, Chicago, IL, USA; f Public Health Sciences, Parkinson School of Health Sciences and Public Health, Loyola University Chicago, Maywood, IL, USA; g Division of Epidemiology and Biostatistics, School of Public Health, Faculty of Health Sciences, University of Cape Town, Cape Town, South Africa

**Keywords:** Sleep health, Socioeconomic status, Circadian rhythms, Sex differences

## Abstract

**Background:**

Risk factors for cardiovascular disease (CVD) and sleep health are well-known to be sex- and race-specific. To build on the established relationship between sleep duration and CVD risk, this cross-sectional study aimed to describe sex-specific associations between CVD risk and other sleep characteristics (sleep quality, sleep timing and sleep onset latency) in low-income adults of African descent.

**Methods:**

Self-reported sleep (Pittsburgh Sleep Quality Index [PSQI], Epworth Sleepiness Scale [ESS], Insomnia Severity Index [ISI]), demographic and lifestyle data were collected in 412 adults (56 % women, 35.0 ± 7.6y, 40 % employed) living in an informal settlement in South Africa. CVD risk was determined using the BMI-modified Framingham 10-year CVD risk formula.

**Results:**

Logistic regression analyses, adjusted for employment, alcohol use and physical activity, indicated that men reporting poor sleep quality (OR: 1.95[95 %CI: 1.07–3.51], *p*=0.025) and earlier bedtimes (0.54[0.39–0.74], *p*<0.001) were more likely to belong to a higher 10-year CVD risk score quintile. Women reporting earlier bedtimes (0.72[0.55–0.95], *p*=0.020) and wake-up times (0.30[0.13–0.73], *p*=0.007), longer sleep-onset latency (1.47[1.43–1.88], *p*=0.003), shorter total sleep times (0.84[0.72–0.98], *p*=0.029), higher PSQI global scores (1.93[1.29–2.90], *p*=0.001) and more moderate to severe symptoms of insomnia (ISI≥15)(3.24[1.04–10.04], *p*=0.016) were more likely to belong to higher 10-year CVD risk score quintile.

**Conclusion:**

In addition to sleep duration, we found that sleep quality, sleep timing and sleep onset latency are additional risk factors for CVD in adults of African descent. Sex-specific differences in the sleep-CVD-risk relationship observed suggests that future studies and recommendations about sleep health in relation to CVD should take sex into account.

## Introduction

Globally, cardiovascular diseases (CVD) are the main contributors to non-communicable disease (NCD) mortality, accounting for 17.9 million deaths annually [1]. African populations are disproportionately affected by CVD with CVD-related deaths contributing to 38 % of all NCD-related deaths in Africa [2]. In the US, African Americans have poorer overall cardiovascular health with higher CVD mortality [3] and significantly higher rates of hypertension compared to their white counterparts [3]. Of particular concern, compared to the global burden of CVD, Africans with CVD are typically younger, predominantly women, and mostly from disadvantaged communities [2].

It is well-established that both metabolic risk factors (i.e. elevated blood pressure, overweight/obesity, hyperglycemia and hyperlipidemia) and poor lifestyle choices and behaviours (i.e. smoking, physical inactivity and excess alcohol use) contribute to the development of NCDs and CVD [4]. Many of these lifestyle factors are considered sexed in that some are more relevant for women and others for men. For example, obesity is predominantly a risk factor for women but smoking still remains a more prominent risk factor for men [4].

Sleep has been identified as a key health behaviour that impacts numerous CVD risk factors [5]. The American Heart Association has recently added sleep as one of the eight essential metrics influencing cardiovascular risks [6]. Sleep is a multidimensional phenomenon which encompasses many different components. Sleep Health is defined by Buysse and colleagues as “*a multidimensional pattern of sleep-wake-fulness, adapted to individual, social and environmental demands, that promotes physical and mental well-being. Good sleep health is characterized by subjective satisfaction, appropriate timing, adequate duration, high efficiency, and sustained alertness during waking hours*” [7]. When looking at overall sleep health and CVD risk, evidence supports the association of adverse health outcomes with both short and long sleep durations such as increased risk for obesity, hypertension, coronary heart disease and type 2 diabetes mellitus [8]. In terms of the other sleep health components, studies are now emerging to show that poor sleep quality is associated with higher risk of hypertension and CVD [9], sleep timing is associated with BMI [10], CVD [11] and overweight/obesity [12], sleep disturbance or fragmentation is associated with CVD [13] and poor sleep regularity is associated with a higher risk for CVD [11,14] and diabetes mellitus [15].

Much of the research investigating associations between sleep and CVD have been conducted in European, Asian or American populations. Very little sleep-related research exists on African-origin populations living outside of the US or the UK. This is relevant since several studies suggest that there may be distinct differences in sleep parameters between European- and African-origin populations [16]. For example, African-Americans have been shown to have poorer sleep continuity and short sleep duration, less slow wave sleep and a greater proportion of non-REM stage 2 sleep than other ethnic groups in the USA [16]. In addition, South African men and women living in low-income settings report much longer sleep durations (8–10 h per night) compared to American populations (6–8 h per night) [17]. Thus the association between sleep characteristics and CVD risk may well differ between socioeconomic contexts.

Given the well-established relationship between sleep duration and CVD risk, this study aims to investigate the relationship between 10-year CVD risk and other sleep characteristics, such as sleep timing, sleep onset latency, sleep quality, daytime sleepiness and insomnia symptom severity in adults of African descent living in a low-income community in South Africa. Furthermore, due to sex differences in CVD risk, the second aim is to determine whether these relationships differ between men and women.

## Participants and methods

### Study design and overview

The Modelling the Epidemiologic Transition Study (METS) is a well-established and ongoing prospective, five-country (Ghana, South Africa, Jamaica, Seychelles, US) cohort study [18]. We report on baseline data collected in the South African cohort of METS-Microbiome, which continues studies in the original longitudinal cohort and is investigating associations between gut microbiota and cardiometabolic disease risk [19]. A full description of the METS-Microbiome study protocol for field staff training, data collection, measurement and laboratory procedures has been published previously [19]. The South African cohort includes both men and women living in Khayelitsha, an informal settlement in Cape Town South Africa. Data collection took place between July 2018 and November 2019. The protocols for the original METS cohort and the present study were approved by the Human Research Ethics Committee of the University of Cape Town, South Africa (Reference numbers: 698/2014 and 155/2020). All participants gave written informed consent and the study strictly adheres to the principles and protocols from the Declaration of Helsinki [20].

### Participants

Four hundred and twelve adults of African descent (178 men [min-max age: 23–55y], 234 women [min-max age: 24–55y]) were recruited and enrolled into the METS-Microbiome study. Participants were excluded at initial enrolment if they were pregnant or lactating (women) or had a condition preventing them from engaging in normal physical activities (e.g. severe osteo- or rheumatoid-arthritis, or lower extremity disability). Two participants who were shift workers were also excluded.

### Demographic and lifestyle questionnaires

The study-specific questionnaires captured the demographic, medical history, medication and supplement use, tobacco and alcohol use, current employment status, highest level of education achieved from participants, as previously described [19]. Participants were classified as current smokers, ex-smokers or non-smokers. They were also classified as alcohol users or non-users, with current number of drinks consumed per week reported for those using alcohol. Current employment status was assessed by the question “Did you do any type of work for pay in the last month?”. Levels of moderate- and vigorous-intensity physical activity (MVPA) was calculated using the Global Physical Activity Questionnaire (GPAQ) [21] and reported as minutes per day. GPAQ is designed to capture physical activity undertaken in different behavioral domains, namely work, transport and recreation/leisure. Within the work and recreation/leisure domains, questions assess the frequency (i.e. number of days per week) and duration (i.e. minutes MVPA per day) of MVPA. Total MVPA per week was calculated as per the GPAQ analysis guide (minutes MVPA per day x number of days per week) [22]. Total MVPA per week was then divided by 7 to get average MVPA per day in minutes. Efforts to address information bias as a result of self-reported data collection included a robust protocol for the collection, measurement and interpretation of information as well as the use of standardized questionnaires and properly calibrated instruments to ensure consistency in data collection [19].

### Sleep characteristics

Self-reported sleep characteristics were assessed using the Pittsburgh Sleep Quality Index (PSQI) questionnaire [23], the Epworth Sleepiness Scale (ESS) [24] and the Insomnia Severity Index (ISI) [25]. Habitual bedtimes (hh:mm), wake-up times (hh:mm), time-in-bed (difference between bedtime and wake-up time (h)), total sleep time (h) and sleep onset latency (min) were all derived from the PSQI. The PSQI global score ranges from 0 to 21 with higher scores indicating poorer sleep quality. Individuals were classified as having poor sleep quality if their PSQI score was >5. We also report the PSQI sub-component score for sleep disturbances, which ranges from 0 to 3 (0 being “no disturbance” and 3 being “severe disturbance”), as this was the only sub-component significantly associated with 10-year CVD risk. The PSQI sub-component of sleep disturbance comprises various dimensions (namely, waking up in the middle of the night or early morning, having to get up to use the bathroom, cannot breathe comfortable, cough or snore loudly, feel too cold, feel too hot, have bad dreams or have pain) which were individually investigated with 10-year CVD risk (Supplementary Table 1). The PSQI questionnaire was administered such that participants reflected on their usual sleep habits during the past month only. Analyses for the other sub-components of the PSQI are presented in the supplementary material (Supplementary Table 2). ESS scores range from 0 to 24 with higher scores indicating greater levels of daytime sleepiness. Participants were categorized as having excessive daytime sleepiness if their ESS scores were >10. ISI scores range from 0 to 28, with higher scores indicating a higher degree of insomnia symptom severity. Participants were classified as having clinical insomnia symptoms (moderate and severe) with ISI scores ≥15, since this is standard practice and enables comparison to existing literature [26]. Given that this population is not a clinical sample, and could rather be classified as a community sample, the lower cut-point of ISI ≥10 was also used. This decision aligns with suggestions that this lower cut-point might be more suitable for community samples [26]. The ISI questionnaire was administered such that participants reflected on their usual sleep habits during the past two weeks only. Mid sleep time was calculated as PSQI wake-up time – (PSQI time-in-bed)/2). Given that employment may play a key role in an individual’s sleep timing, duration and quality, descriptive sleep characteristics between employed and unemployed men and women are presented in Supplementary Table 3.

### Anthropometry

Weight (kg), height (cm), waist and hip circumferences (cm) were measured according to the METS standard procedures. Body mass index (BMI) was calculated as weight/height^2^ (kg/m^2^). Participants were classified as normal weight (BMI: ≥18.5 but <24.9 kg/m^2^) overweight (BMI: ≥25 kg/m^2^ but <30 kg/m^2^), or obese (BMI: ≥30 kg/m^2^) and having a high waist circumference (≥102 cm in men and ≥88 cm in women).

### Clinical measurements

Resting systolic (SBP) and diastolic (DBP) blood pressure were measured in triplicate on two separate occasions (Omron HEM-747Ic, Omron Healthcare, Bannockburn, IL, USA) as previously described [19]. Participants were classified as having elevated BP if either their measured SBP was ≥130 mmHg, their DBP was ≥85 mmHg, they reported being diagnosed with hypertension or they were on antihypertensive medication. Following an overnight fast, fasting capillary plasma glucose concentration was determined using the finger stick method (Accu-check Aviva, Roche). Participants were classified as having high fasting plasma glucose if their measured glucose was ≥5.6 mmol/L or they were using medication to treat diabetes.

### 10-year CVD risk

CVD risk was assessed using the BMI-modified sex-specific Framingham 10-year CVD risk score, which substitutes laboratory values of measured total cholesterol and HDL-C with measured BMI [27]. The seven risk factors include: age, sex, measured SBP, treatment of hypertension, diagnosed diabetes, measured BMI and smoking status. This modified score substitutes measured cholesterol and HDL-cholesterol with measured BMI and estimates the risk of developing CVD within the next 10 years. The decision to use the BMI-modified Framingham 10-year CVD risk score was based on the previously identified “triglyceride paradox” present in individuals of African descent [28]. This paradox describes how, even though insulin resistance, CVD and type 2 diabetes are associated with hypertriglyceridemia, individuals of African descent with these conditions usually have normal triglyceride levels.

### Data and statistical analyses

Data are presented as mean ± standard deviation, median (interquartile range), frequency counts (%), relative risk ratios or odds ratios (OR) with 95 % confidence intervals (CI). The Shapiro-Wilk test was used to test for normality. Between group comparisons were made using one-way analysis of variance or covariance, Kruskal-Wallis tests, Chi-Squared or Fisher’s Exact tests. Since the 10-year CVD risk score was not normally distributed and could not be transformed, the score was coded into quintiles (reference quintile 1). Ordered logistic regression analyses examined associations between self-reported sleep variables (independent) and 10-year CVD risk score quintiles (dependent). The continuous version of the independent variables (i.e. all sleep variables) were used.

We ensured that our ordered logistic regression model met all key assumptions, which includes meeting the proportional odds assumption. To confirm this, we utilized the Brant test, a statistical method specifically designed to assess the validity of the proportional odds assumption. We only present data for models which met the assumption. Covariates included in the fully adjusted model for 10-year CVD risk were alcohol consumption, MVPA and employment status. Age, sex, BMI and smoking status were not included as covariates as they are factors included in the 10-year CVD risk score. Data were analysed using Stata V15.1 (StataCorp, Texas, USA). Since, only six participants (1.5 %) reported any previous CVD events and only two participants (0.5 %) reported being diagnosed with depression, these variables were not statistically modelled with 10-year CVD risk scores.

## Results

### Participant characteristics

The descriptive characteristics of the participants are presented in Table 1. Disproportionately more women than men were classified as being overweight (*p* < 0.001) or obese (*p* < 0.001) or having a high waist circumference (*p* < 0.001). Women also had higher BMIs (*p* < 0.001) and engaged in less MVPA (*p* < 0.001) than men. More men presented with higher SBP (*p* < 0.001), fasting blood glucose levels (*p* = 0.001) and 10-year CVD risk scores (*p* < 0.001) compared to women. More men were smokers (*p* < 0.001), consumed alcohol (*p* < 0.001) and consumed more alcoholic units per week (*p* < 0.001) than women. Unemployment was higher among the women (*p* < 0.001). No participants reported taking any sleep medication.

### Self-reported sleep characteristics

Self-reported sleep characteristics for both men and woman are presented in Table 2. Among all participants, 28 (6.8 %) reported a total sleep time of <7 h per night, 245 (59.5 %) reported 7–9 h per night and 139 (33.7 %) reported >9 h per night. Sleep timing and duration were similar between the men and women, however, men reported taking longer to fall asleep compared to women (*p* = 0.011) and women experienced more moderate sleep disturbances than men (*p* = 0.003).

### Sex-specific associations between self-reported sleep characteristics and CVD risk score quintiles

Separate ordered logistic regression models for men and women testing associations between 10-year CVD risk score and self-reported sleep characteristics are also shown in Table 3. In the fully adjusted models, men who reported earlier bedtimes (0.54 (95 %CI: 0.39–0.74), *p* < 0.001) were more likely to belong to a higher 10-year CVD risk score quintile compared to those with later bedtimes. Men who were classified as having poor sleep quality were 1.95 (1.07–3.51, *p* = 0.025) times more likely to belong to a higher 10-year CVD risk score quintile compared to those with good sleep quality. Although the overall PSQI sleep disturbance model was not significant, when investigating the PSQI sleep disturbance sub-component dimensions, men who reported having pain three or more times a week were 2.79 (1.11–7.03, *p* = 0.029) times more likely to belong to a higher 10-year CVD risk score quintile compared to those who reported no pain (Supplementary Table 1).

Among the women, in the fully adjusted models, those reporting earlier bedtimes (0.72 (0.55–0.95), *p* = 0.020) and wake-up times (0.30 (0.13–0.72), *p* = 0.007) were more likely to belong to a higher 10-year CVD risk score quintile than those reporting later bedtimes and wake-up times. Those women who reported a longer sleep-onset latency were 1.47 (1.43–1.88, *p* = 0.003) times more likely to belong to a higher 10-year CVD risk score quintile than women who reporting a short sleep-onset latency. Those with shorter total sleep times (0.84 (0.72–0.98), *p* = 0.029) were more likely to belong to a higher 10-year CVD risk score quintile than those with longer total sleep times. Women with higher PSQI global scores were 1.93 (1.29–2.90, *p* = 0.001) times more likely to belong to a higher 10-year CVD risk score quintile. Women classified as having moderate to severe insomnia symptoms (ISI ≥10 and ISI≥15) were 2.25 (1.15–4.37, *p* = 0.016) and 3.24 (1.04–10.04, *p* = 0.042) times more likely to belong to a higher 10-year CVD risk score quintile. Although the overall PSQI sleep disturbance model was not significant, when investigating the PSQI sleep disturbance sub-component dimensions among the women, those who reported waking up in the middle of the night or early morning once or twice a week or three or more times a week were 2.39 (1.30–4.37, *p* = 0.005) and 2.73 (1.38–5.37, *p* = 0.004) times more likely to belong to a higher 10-year CVD risk score quintile compared to those who reported not waking up early (Supplementary Table 1). Similarly, women who reported feeling hot once or twice a week were 1.88 (1.08–3.28, *p* = 0.027) times more likely to belong to a higher 10-year CVD risk score quintile compared to those who reported not feeling hot (Supplementary Table 1).

## Discussion

We confirm the long self-reported sleep duration (time-in-bed: 9.24 ± 1.59 h) observed in previous South African studies among individuals of African descent [17,29]. In the present study, among the men, earlier bedtimes and poorer sleep quality were associated with an increased 10-year CVD risk. Among the women, earlier bedtimes and wake-up times, taking longer to fall asleep, shorter total sleep time, poor sleep quality and greater severities of insomnia symptoms were all independently associated with an increased 10-year CVD risk. Three main factors could likely explain these findings: i) the populations sleep environment, ii) the neighbourhood environment and iii) the poor overall health of this population.

Earlier bedtimes were associated with higher 10-year CVD risk score quintiles among the men and the women. Later bedtime are commonly thought as being a negative sleep behaviour since later sleep timing is often associated with poorer cardiovascular health [30], however, we have found the opposite. We speculate that these participants are trying to fall asleep too early which might be out of phase with their endogenous circadian rhythms. It is well-established that disruption to our body’s natural circadian rhythms can be detrimental to cardiovascular health [30]. Thus, whether early bedtimes are associated with circadian misalignment in this population should be assessed in future studies. In addition, it may be that the general physical health of this population is relatively poor, therefore they are going to bed earlier because they may feel unwell. One could speculate that this is similar to long sleep (i.e. sleep > 10 h) being associated with adverse health outcomes not because of the sleep itself, but because of underlying conditions that change sleep behaviour [31]. While this finding contrasts with what the global North has shown regarding later bedtimes and poorer overall health, it reinforces why we need to examine questions around the relationship between sleep and CVD risk in more diverse populations to understand population-specific CVD risk factors.

Poor sleep quality was associated with higher 10-year CVD risk score quintiles among the men and women. Among women specifically, waking up in the middle of the night or waking too early in the morning, as well as feeling too hot was associated with higher 10-year CVD risk score quintiles. This finding could be explained by the fact that these individuals reside in a township where they live in houses which are over-crowded, have poor ventilation, are often noisy and have poor safety or security. Typically, these temporary homes comprise only one or two rooms, with a housing density of 4 (range: 3–6) individuals per home (38–84m^4^), leaving occupants with little privacy or quiet spaces for sleep. Many of these factors have been previously associated with worse sleep quality [32] and worse CVD health [33]. Two other explanations for this finding could be that sleep disturbances may interrupt the physiological recovery function of sleep, specifically where the disturbances do not allow for the body to fully decrease sympathetic nervous system (SNS) activity thereby preventing the body from resting, and therefore might lead to poorer cardiovascular health [33]. Alternatively, inflammation and inflammatory cytokines are increased in individuals with cardiovascular diseases [34]. Therefore, one could alternatively speculate that poorer sleep quality may be leading to higher levels of inflammation with subsequent poorer cardiovascular health in this population, especially among the women where the obesity rates are very high. Altogether, this finding strengthens the need to include sleep quality as an important indicator when assessing CVD risk among low-income populations such as this, especially in women.

Longer PSQI sleep-onset latencies as well as presenting with more moderate to severe insomnia symptoms were also associated with higher 10-year CVD risk scores among the women. Stress-related psychological factors are central to the pathogenesis and maintenance of insomnia, mostly due to increased and inappropriate SNS activity during sleep [35]. Additionally, overactivity of the SNS has long been recognized to be a major mediator in the relationship between stress and CVD [36]. In addition to the poor sleep environment mentioned above, the crime and violence rates (specifically contact crimes such as murder, assault and sexual offences) in this township are well above the national average [37]. Given that women in this neighbourhood are often the victims of crime, it is entirely plausible that hyperarousal or overactivation of the SNS may play a key role in the difficulties falling asleep. Previous work by Mellman et al. (2018) in urban-residing African Americans reported associations between indicators of stressful environments and increased sympathetic, decreased parasympathetic nervous system activity (i.e. hyperarousal) during sleep [38]. It is also worth noting that in this particular study greater effects were observed in women, particularly for those with a higher degree of exposure to violence [38]. Another study done in South African female survivors of sexual assault found that PTSD-diagnosed individuals felt safer sleeping in the laboratory than in their home environments and that they experienced fewer sleep disruptions during a night of laboratory sleep than they reported experiencing during sleep at home [39]. Neighborhood environment aside, the prevalence of sleep disorders (specifically insomnia) and the risk of developing psychiatric problems (such as depression and anxiety [40] which often increase the risk of insomnia) are more prevalent in women compared to men [40]. Qualitative studies using interviews to explore perceptions around sleep and the home or neighbourhood environment in this population are needed to assess how neighborhood safety and stress might contribute to poor sleep quality.

Among the women, earlier wake-up times were associated with higher 10-year CVD risk score quintiles. This is similar to what has previously been shown in individuals of European descent where waking up early increased risk of mortality from CVD [41]. Two important cultural aspects of this society may help explain this finding. Firstly, the women in the community are often the caregivers and the ones who need to get the children, grandchildren or extended family ready for school or work, meaning that their wake-up times may be earlier than preferred. One might hypothesize that those women who wake up earlier may subsequently have a shorter overall sleep opportunity. Secondly, due to the low socioeconomic nature of this population it means that the individuals who are employed may have to wake up very early in order to catch public transport and commute to get to work on time. Indeed, this is what we see when looking at the sleep characteristics of employed versus unemployed women; employed women wake up around one hour earlier than unemployed women. Additionally, employed women had a shorter time-in-bed and total sleep time with earlier average wake-up times.

Among the women, shorter total sleep times were associated with increased 10-year CVD risk. Intriguing is that, even though the women are spending around 9 h (±1.5 h) in bed (i.e. the upper limit of the recommended guidelines of 7–9h [42]), the relationship between sleep and CVD risk still exists. This relationship is not present in the men, but one could argue that it seems the women have poorer overall sleep health compared to the men. While on the surface it looks that their sleep duration is sufficient, they are potentially accumulating less sleep overall as their sleep is more fragmented as described above. Given the limitation of self-report, however, future research that objectively measures sleep duration through actigraphy is needed to shed more light on this aspect of sleep.

Our study is not without limitations, the main one being that sleep measured in this study is self-reported, as opposed to objectively measured, and limited in time to the past month or past 2 weeks for PSQI and ISI tools, respectively. It is possible that individuals may over- or under-estimate their sleep duration and quality. The prevalence of obstructive sleep apnea (OSA) or other sleep and mental health disorders was not assessed in this study, but may have influenced the nature of sleep in this population and thus the sleep-CVD risk relationships. This is something that needs to be included in future studies. Finally, our data are cross-sectional, and we are therefore not able to speculate any cause and effect relationships between self-reported sleep characteristics and 10-year CVD risk scores.

In conclusion, while the sleep health between sexes in this population may be different due to crime, employment/unemployment or cultural aspects of the society, for example, it may also be due to the drastically different primary CVD risk factors (i.e. smoking status and obesity) between men and women. Overall, we have shown that in addition to thinking about only short or long sleep duration as a risk factor for CVD we should also consider other components of sleep health such as sleep timing, sleep onset latency and sleep quality when assessing an individuals’ CVD health. Although we have looked at these sleep factors individually, we know that sleep is actually multifaceted with sleep variables being distinct but often interrelated. Future use of a multidimensional sleep health score to assess and understand all variables of sleep as they related to CVD may be more important for better prevention and treatment of CVD.

## Supplementary Material

1

## Figures and Tables

**Table 1:** Descriptive characteristics of participants stratified by sex.

	Men (*n*=178)	Women (*n*=234)	*p value*
Age (y)	36 (31 – 42)	34 (29 – 42)	*p = 0.178*
BMI (kg/m^2^)	21.5 (19.4 – 24.3)	32.7 (26.7 – 38.2)	***p < 0.001***
Overweight (count,%)	37 (20.8)	190 (81.2)	***p < 0.001***
Obese (count,%)	10 (5.6)	141 (60.3)	***p < 0.001***
Waist circumference (cm)	79.6 (73.8 – 87.1)	98.1 (88.5 – 112.0)	***p < 0.001***
High waist circumference (count,%)	11 (6.2)	176 (75.2)	***p < 0.001***
SBP (mmHg)	121 (110 – 131)	111 (103 – 122)	***p < 0.001***
DBP (mmHg)	74 (66 – 82)	73 (66 – 80)	*p = 0.492*
Elevated BP (count,%)	62 (34.8)	66 (28.2)	*p = 0.150*
Fasting glucose (mmol/L)	5.0 (4.6 – 5.6)	4.7 (4.4 – 5.2)	***p = 0.001***
High glucose (count,%)	7 (3.9)	8 (3.4)	*p = 0.789*
10-year CVD risk score (%)	3.9 (1.9 – 6.9)	2.0 (1.0 – 4.4)	***p < 0.001***
Highest degree of formal education			*p = 0.238*
*None or Primary (count,%)*	114 (64.1)	144 (61.6)	
*Secondary (count,%)*	47 (26.4)	75 (32.0)	
*Tertiary (count,%)*	16 (8.9)	14 (5.9)	
Employed (count,%)	95 (53.4)	69 (29.5)	***p < 0.001***
MVPA (min/day)	51.4 (22.9 – 98.6)	18.6 (8.6 – 42.9)	***p < 0.001***
Smoking status			***p < 0.001***
*Smoker (count,%)*	126 (70.8)**	42 (17.9)**	
*Non-smoker (count,%)*	38 (21.3)**	188 (80.3)**	
*Ex-smoker (count,%)*	11 (6.2)*	5 (2.1)*	
Alcohol user (count,%)	168 (94.4)	171 (73.1)	***p < 0.001***
Alcohol (no. drinks per week)	16.0 (0 – 36)	0.0 (0 – 7)	***p < 0.001***

Data are presented as median (interquartile range) or count (%).

P values represent differences between men and women.

* *p* < 0.050 and

** *p* < 0.001 represent post-hoc analyses using Fisher’s exact tests.

BMI: body mass index; BP: blood pressure; CVD: cardiovascular disease; DBP: diastolic blood pressure; MVPA: moderate- and vigorous-intensity physical activity; SBP: systolic blood pressure.

**Table 2:** Self-reported sleep characteristics for participants stratified by sex.

	Men (*n*=178)	Women (*n*=234)	*p value*
PSQI Bedtime (hh:mm)	22:00 (21:00 – 22:00)	21:30 (21:00 – 22:00)	*p = 0.620*
PSQI Wake-up time (hh:mm)	07:00 (06:00 – 08:00)	07:00 (06:00 – 08:00)	*p = 0.875*
PSQI Time-in-bed (h)	9.16 ± 1.65	9.30 ± 1.55	*p = 0.338*
PSQI Total sleep time (h)	8.55 ± 1.57	8.79 ± 1.53	*p = 0.131*
PSQI Midpoint of sleep (hh:mm)	02:25 ± 00:55	02:00 ± 00:55	*p = 0.724*
PSQI SOL (min)	30 (15 – 30)	20 (10 – 30)	***p = 0.011***
SOL >30 min (count,%)	41 (23.03)	40 (17.09)	*p = 0.131*
PSQI global score	4 (3 – 6)	4 (3 – 6)	*p = 0.560*
Poor sleep quality (count,%)	54 (30.3)	67 (28.6)	*p = 0.679*
PSQI: Sleep disturbance (count,%)			***p = 0.003***
*None*	5 (2.8)	8 (3.4)	
*Mild*	109 (61.2)	110 (47.0)	
*Moderate*	55 (30.9)**	112 (46.9)**	
*Severe*	8 (4.5)	4 (1.7)	
ESS score	6 (3 – 10)	7 (4 – 11)	*p = 0.129*
Excessive daytime sleepiness (ESS>10) (count,%)	49 (27.5)	80 (34.3)	*p = 0.151*
ISI score	2 (1 – 5)	3 (1 – 6)	*p = 0.089*
Moderate to severe insomnia symptoms (ISI≥15) (count,%)	7 (3.9)	10 (4.3)	*0.531*
Moderate to severe insomnia symptoms (ISI≥10) (count,%)	22 (12.4)	32 (13.7)	*p = 0.399*

Data are presented as mean ± standard deviation, median (interquartile range) or count (%).

P values represent comparisons between men and women.

* *p* < 0.050 and

** *p* < 0.001 represent post-hoc analyses using Fisher’s exact tests.

ESS: Epworth Sleepiness Scale; ISI: Insomnia Severity Index; PSQI: Pittsburgh Sleep Quality Index; SOL: sleep onset latency.

**Table 3:** Fully adjusted ordered logistic regression models exploring the associations between 10-year CVD risk score quintile (dependent variable) and self-reported sleep characteristics (independent variables) stratified by sex.

	Men		Women	
	OR (95 % CI)	*p value*	OR (95 % CI)	*p value*
Bedtime	0.54 (0.39 – 0.74)	**<0.001**	0.72 (0.55 – 0.95)	**0.020**
*Overall model*	*n=177, LR chi^2^=22.04, p = 0.002*		*n=233, LR chi^2^=11.76, p = 0.016*	
Wake-up time	0.45 (0.18 – 1.14)	0.093	0.30 (0.13 – 0.73)*	**0.007**
*Overall model*	*n=176, LR chi^2^=9.71, p = 0.045*		*n=233, LR chi^2^=13.47, p = 0.009*	
Time-in-bed	1.05 (0.89 – 1.23)	0.532	0.906 (0.78 – 1.06)	0.216
*Overall model*	*n=176, LR chi^2^=8.04, p = 0.090*		*n=232, LR chi^2^=7.65, p = 0.105*	
Total sleep time	1.01 (0.85 – 1.19)	0.927	0.84 (0.72 – 0.98)*	**0.029**
*Overall model*	*n=176, LR chi^2^=6.89, p = 0.141*		*n=233, LR chi^2^=11.18, p = 0.025*	
SOL	1.25 (0.93 – 1.69)	0.139	1.47 (1.43 – 1.88)*	**0.003**
*Overall model*	*n=176, LR chi^2^=9.11, p = 0.0584*		*n=233, LR chi^2^=15.55, p = 0.004*	
SOL > 30 min (v SOL < 30 min)	0.76 (0.42 – 1.37)	0.366	1.27 (0.78 – 2.07)	0.344
*Overall model*	*n=176, LR chi^2^=7.98, p = 0.092*		*n=232, LR chi^2^=7.26, p = 0.122*	
PSQI global score	1.45 (0.95 – 2.22)	0.085	1.93 (1.29 – 2.90)*	**0.001**
*Overall model*	*n=176, LR chi^2^=9.97, p = 0.041*		*n=233, LR chi^2^=116.65, p = 0.002*	
Poor sleep quality (v good sleep quality)	1.95 (1.07 – 3.51)*	**0.025**	1.41 (0.85 – 2.35)	0.176
*Overall model*	*n=176, LR chi^2^=12.00, p = 0.017*		*n=233, LR chi^2^=8.08, p = 0.088*	
PSQI disturbance				
*Mild v None*	2.12 (0.43 – 10.35)	0.353	3.89 (0.93 – 16.16)	0.063
*Moderate v None*	3.80 (0.74 – 19.45)	0.108	4.51 (1.08 – 18.68)*	0.038
*Severe v None*	1.78 (0.23 – 13.77)	0.579	7.31 (0.61 – 88.78)	0.117
*Overall model*	*n=176, LR chi^2^=12.44, p = 0.053*		*n=233, LR chi^2^=11.43, p = 0.076*	
ESS	0.89 (0.69 – 1.15)	0.399	1.07 (0.86 – 1.34)	0.523
*Overall model*	*n=176, LR chi^2^=7.87, p = 0.096*		*n=232, LR chi^2^=6.77, p = 0.148*	
Excessive daytime sleepiness	0.76 (0.43 – 1.37)	0.366	1.26 (0.78 – 2.07)	0.344
*Overall model*	*n=176, LR chi^2^=7.98, p = 0.092*		*n=232, LR chi^2^=7.26, p = 0.123*	
ISI	1.00 (0.94 – 1.07)	0.955	1.05 (1.00 – 1.11)*	**0.038**
*Overall model*	*n=176, LR chi^2^=6.88, p = 0.142*		*n=232, LR chi^2^=10.66, p = 0.031*	
Moderate to severe insomnia symptoms (ISI≥15)	1.39 (0.41 – 4.67)	0.591	3.24 (1.04 – 10.04)	**0.042**
*Overall model*	*n=177, LR chi^2^=7.17, p = 0.013*		*n=232, LR chi^2^=10.50, p = 0.014*	
Moderate to severe insomnia symptoms (ISI≥10)	0.95 (0.43 – 2.09)	0.900	2.25 (1.15 – 4.37)*	**0.016**
*Overall model*	*n=177, LR chi^2^=6.90, p = 0.141*		*n=232, LR chi^2^=12.15, p = 0.016*	

Models were determined using ordered logistic regressions and data are presented as odds ratios (OR) with 95 % confidence intervals (CI). Models were adjusted for alcohol, MVPA and employment.

* p < 0.05,

** p < 0.001.

CVD: cardiovascular disease, SOL: sleep onset latency, PSQI: Pittsburg Sleep Quality Index, ESS: Epworth Sleepiness Scale, ISI: insomnia severity index; MVPA: moderate-vigorous physical activity.
