# Supplementary material for: Sex-specific associations between self-reported sleep characteristics and 10-year cardiovascular disease risk in men and women of African descent living in a low socioeconomic status environment
Source: Sleep Epidemiol. Author manuscript; Available in PMC 2025 Jan 10. (PMC11720418; doi:10.1016/j.sleepe.2024.100091)
Supplement: 1 [file NIHMS2041526-supplement-1.docx]

**Supplementary Table 1. Fully adjusted ordered logistic regression models exploring the associations between 10-year CVD risk score quintile (dependent variable) and the PSQI sleep disturbance dimensions (independent variables) stratified by sex.**

|  | **Men** | | **Women** | |
| --- | --- | --- | --- | --- |
|  | OR (95% CI) | *p value* | OR (95% CI) | *p value* |
| **Waking up in the middle of the night** |  |  |  |  |
| *<1 time a week v None* | 0.92 (0.32 – 2.45) | 0.864 | 1.22 (0.55 – 2.70) | 0.625 |
| *1-2 times a week v None* | 1.29 (0.67 – 2.47) | 0.439 | 2.39 (1.30 – 4.37)* | **0.005** |
| *≥3 times a week v None* | 1.37 (0.65 – 2.89) | 0.406 | 2.73 (1.38 – 5.37)* | **0.004** |
| *Overall model* | *n=177, LR chi^2^=8.11, p=0.229* | | *n=233, LR chi^2^=18.55, p=0.005* | |
| **Getting up for the bathroom** |  |  |  |  |
| *<1 time a week v None* | 0.46 (0.11 – 1.88) | 0.282 | 0.57 (0.14 – 2.19) | 0.415 |
| *1-2 times a week v None* | 1.07 (0.55 – 2.07) | 0.844 | 1.07 (0.56 – 2.04) | 0.829 |
| *≥3 times a week v None* | 1.65 (0.84 – 3.32) | 0.142 | 0.92 (0.49 – 1.69) | 0.782 |
| *Overall model* | *n=177, LR chi^2^=11.45, p=0.021* | | *n=233, LR chi^2^=7.52, p=0.010* | |
| **Cannot breathe comfortably** |  |  |  |  |
| *<1 time a week v None* | 1.32 (0.45 – 3.82) | 0.606 | *0.89 (0.29 – 2.70)* | *0.845* |
| *1-2 times a week v None* | 1.52 (0.65 – 3.53) | 0.335 | *0.83 (0.35 – 1.96)* | *0.680* |
| *≥3 times a week v None* | 0.44 (0.0 – 2.16) | 0.315 | *3.24 (0.86 – 12.21)* | *0.082* |
| *Overall model* | *n=177, LR chi^2^=9.02, p=0.016* | | *n=233, LR chi^2^=9.59, p=0.013* | |
| **Cough or snore loudly** |  | |  | |
| *<1 time a week v None* | 0.84 (0.17 – 4.01) | 0.826 | 1.15 (0.38 – 3.41) | 0.797 |
| *1-2 times a week v None* | 1.20 (0.62 – 2.72) | 0.479 | 1.47 (0.77 – 2.82) | 0.239 |
| *≥3 times a week v None* | 1.11 (0.46 – 2.66) | 0.818 | 1.06 (0.58 – 1.93) | 0.845 |
| *Overall model* | *n=177, LR chi^2^=7.47, p=0.014* | | *n=233, LR chi^2^=8.02, p=0.011* | |
| **Feel too hot** |  |  |  |  |
| *<1 time a week v None* | 1.36 (0.58 – 3.19) | 0.469 | 1.02 (0.46 – 2.22) | 0.958 |
| *1-2 times a week v None* | 1.33 (0.71 – 2.49) | 0.372 | 1.88 (1.08 – 3.28)* | **0.027** |
| *≥3 times a week v None* | 1.90 (0.76 – 4.71) | 0.166 | 1.24 (0.58 – 2.62) | 0.572 |
| *Overall model* | *n=177, LR chi^2^=9.26, p=0.159* | | *n=233, LR chi^2^=11.32, p=0.016* | |
| **Feel too cold** |  | |  | |
| *<1 time a week v None* | 1.92 (0.66 – 5.60) | 0.230 | 0.78 (0.36 – 1.69) | 0.530 |
| *1-2 times a week v None* | 1.32 (0.67 – 2.58) | 0.418 | 1.43 (0.84 – 2.48) | 0.201 |
| *≥3 times a week v None* | 0.62 (0.23 – 1.65) | 0.345 | 1.52 (0.73 – 3.15) | 0.258 |
| *Overall model* | *n=175, LR chi^2^=10.92, p=0.091* | | *n=232, LR chi^2^=9.41, p=0.012* | |
| **Have bad dreams** |  | |  | |
| *<1 time a week v None* | 1.21 (0.54 – 2.70) | 0.639 | 0.86 (0.43 – 1.72) | 0.664 |
| *1-2 times a week v None* | 1.04 (0.56 – 1.95) | 0.895 | 1.03 (0.59 – 1.080) | 0.897 |
| *≥3 times a week v None* | 1.91 (0.70 – 5.17) | 0.204 | 1.03 (0.50 – 2.11) | 0.935 |
| *Overall model* | *n=175, LR chi^2^=9.06, p=0.017* | | *n=230, LR chi^2^=6.37, p=0.383* | |
| **Have pain** |  |  |  |  |
| *<1 time a week v None* | 1.63 (0.69 – 3.81) | 0.262 | 0.98 (0.47 – 2.05) | 0.963 |
| *1-2 times a week v None* | 1.40 (0.72 – 2.72) | 0.321 | 1.67 (0.96 – 2.92) | 0.069 |
| *≥3 times a week v None* | 2.79 (1.11 – 7.03)* | **0.029** | 1.17 (0.55 – 2.49) | 0.683 |
| *Overall model* | *n=177, LR chi^2^=12.52, p=0.023* | | *n=233, LR chi^2^=9.83, p=0.013* | |

Models were determined using ordered logistic regressions and data are presented as odds ratios (OR) with 95% confidence intervals (CI). Models were adjusted for alcohol, MVPA and employment. **p<0.05. “None” refers to not during the past month.*

CVD: cardiovascular disease, PSQI: Pittsburg Sleep Quality Index, MVPA: moderate-vigorous physical activity

**Supplementary Table 2. Fully adjusted ordered logistic regression models exploring the associations between CVD risk score quintile (dependent variable) and self-reported PSQI sleep variables (independent variables) stratified by sex.**

|  | **Men** | | **Women** | |
| --- | --- | --- | --- | --- |
|  | OR (95% CI) | *p value* | OR (95% CI) | *p value* |
| **PSQI sleep efficiency** | 1.32 (0.58-2.16) | 0.708 | 1.47 (0.88-2.44) | 0.135 |
| *Overall model* | *n=175, LR chi^2^=9.36, p=0.052* | | *n=233, LR chi^2^=6.34, p=0.175* | |
| **PSQI sleep medication** | 0.87 (0.44-1.69) | 0.679 | 1.25 (0.78-2.00) | 0.347 |
| *Overall model* | *n=176, LR chi^2^=11.23, p=0.024* | | *n=233, LR chi^2^=4.87, p=0.301* | |
| **PSQI daytime dysfunction** | 1.03 (0.75-1.43) | 0.839 | 1.18 (0.89-1.57) | 0.236 |
| *Overall model* | *n=175, LR chi^2^=9.26, p=0.055* | | *n=233, LR chi^2^=5.40, p=0.249* | |

CVD: cardiovascular disease; PSQI: Pittsburgh Sleep Quality Index

**Supplementary Table 3. Self-reported sleep characteristics between employed and unemployed men and women.**

|  | Employed | | Unemployed | |
| --- | --- | --- | --- | --- |
|  | **Men** | **Women** | **Men** | **Women** |
| PSQI Bedtime (hh:mm) | 22:00 (21:00-22:00) | 21:30 (21:00-22:00) | 22:00 (21:00-22:00 | 21:30 (21:00-22:00) |
| PSQI Wake-up time (hh:mm) | 07:00 (06:00-07:30) | 06:00 (06:00-07:00) | 07:00 (06:00-08:00) | 07:00 (06:00-08:00) |
| PSQI Time-in-bed (h) | 9.0 (8.0-10.0) | 9.0 (8.0-10.0) | 9.5 (8.3-10.5) | 9.5 (8.5-10.5) |
| PSQI Total sleep time (h) | 8.5 (7.5-9.5) | 8.3 (7.0-9.5) | 9.0 (7.8-9.8) | 9.0 (8.0-10.0) |
| PSQI Mid point of sleep (hh:mm) | 2:00 ± 00:49 | 02:00 ± 00:50 | 2:30 ± 1.11 | 2:09 ± 00:57 |
| PSQI SOL (min) | 20 (15-30) | 15 (10-30) | 30 (15-30) | 20 (10-30) |

*Data are presented as mean ± standard deviation, median (interquartile range) or count (%).*

PSQI: Pittsburgh Sleep Quality Index; SOL: sleep onset latency.
